# The Model Repository of the Models of Infectious Disease Agent Study

**DOI:** 10.1109/TITB.2007.910354

**Published:** 2008-07-10

**Authors:** Phillip C. Cooley, D. Roberts, V. D. Bakalov, S. Bikmal, S. Cantor, T. Costandine, L. Ganapathi, B. J. Golla, G. Grubbs, C. Hollingsworth, S. Li, Y. Qin, William Savage, D. Simoni, E. Solano, D. Wagener

**Affiliations:** 1 RTI International Research Triangle ParkNC 27709 USA; 2 Optimacy Corporation Colorado SpringsCO 80905 USA

**Keywords:** Data management, data warehouse, epidemiological disease models, model repository (MREP), relational database

## Abstract

The model repository (MREP) is a relational database management system (RDBMS) developed under the auspices of models of infectious disease agent study (MIDAS). The purpose of the MREP is to organize and catalog the models, results, and suggestions for using the MIDAS and to store them in a way to allow users to run models from an access-controlled disease MREP. The MREP contains source and object code of disease models developed by infectious disease modelers and tested in a production environment. Different versions of models used to describe various aspects of the same disease are housed in the repository. Models are linked to their developers and different versions of the codes are tied to Subversion, a version control tool. An additional element of the MREP will be to house, manage, and control access to a disease model results warehouse, which consists of output generated by the models contained in the MREP. The result tables and files are linked to the version of the model and the input parameters that collectively generated the results. The result tables are warehoused in a relational database that permits them to be easily identified, categorized, and downloaded.

## I. Introduction

This paper describes the model repository (MREP) of models of infectious disease agent study (MIDAS), which is a tool developed to organize and catalog the models, results, and suggestions for using results of the MIDAS and store them in a relational database for future use and reference. The database is a repository of epidemiological-based infectious disease models and their derivatives (e.g., inputs, parameters, and outputs). These products will be stored to allow easy retrieval via a query method. This section gives a brief explanation of the MIDAS and the rationale for the MREP, and identifies other related tools in the literature. Section II presents the architecture and design of the MREP. The MREP's key design elements include the ability to link all related model components together and to include a version control tool as part of the relational data base management system (RDBMS) data model. Section III describes the content of the MREP, including the information about the MIDAS models and the studies they comprise. Section IV summarizes key features of the MREP and discusses future enhancements.

### A. Midas

The MIDAS is a research partnership between the National Institutes of Health (NIH) and the scientific community to develop computational models for policymakers, public health workers, and researchers to help them make better-informed decisions about emerging infectious diseases—both man made and naturally occurring. The MIDAS researchers are working to develop models that can assist the public health community understand how best to respond during outbreaks and epidemics. The MIDAS consists of seven research groups and one centralized informatics group.

#### 1. MIDAS Objectives

The MIDAS Research Groups develop epidemiological models that represent host-pathogen relationships, disease epidemiology and forecasting systems, and pandemic response strategies. They also focus on information-driven research rather than hypothesis-driven investigations. The MIDAS model developers use real or simulated data available through the MIDAS Web site. As a collaborative network of scientists, the MIDAS investigates using computational and mathematical models that will prepare the nation to respond to outbreaks of infectious diseases by providing policymakers and public health officials with reliable and timely information that can be used to prepare for infectious disease outbreaks.

#### 2. Epidemiological Models

Epidemic models represent powerful tools for gaining insight into how the dynamics of an epidemic are affected by interventions. In order to understand and control the spread of pathogens, it is essential to establish some of the key parameters associated with disease transmission. Fundamental to the dynamics of an epidemic are two quantities: the basic reproduction number (R0) and the generation time (Tg) of the pathogen [1]. The R0 is the average number of secondary cases produced by each primary case at the start of an epidemic in a previously unaffected population. The Tg is the average time between infection of index cases and the secondary cases they produce. The R0 is a measure of the transmissibility of the strain in the population, and largely determines the proportion of the population that will be infected in a pandemic. The ratio R0/Tg is a measure of an epidemic's rate of growth.

Many epidemiological models are based on a compartmental, Sampling Importance Resampling (SIR) framework; the host population is partitioned into those that are susceptible, infected, or immune to a particular pathogen. These models assume that the rate at which new infections are acquired is proportional to the number of encounters between susceptible and infected individuals, and leads to an effective reproductive ratio that depends on a threshold density of susceptibles. Thus, the models depend not only on parameters intrinsic to the disease such as latent and infectious periods, but also on contacts between infectious and susceptible hosts. Historically, structuring the population at risk into compartments permits subpopulations of varying risks to be represented. Compartmental models of this kind implicitly assume that the host population is well mixed, such that the probability of infection is equal for all.

However, social network structures are clearly not always well mixed, and the complexities of host interactions may have profound implications for the interpretation of epidemiological models and clinical data. To overcome the simplifying assumption of classical transmission models, a new type of model is gaining recognition. Agent-based models (ABMs) represent an important new approach for describing interacting heterogeneous agents. The heterogeneous aspect of agents enables more sophisticated and complex environments to be described by ABMs approaches. Also, it is typical to introduce a geospatial dimension into the framework so that both time and geographical patterns are represented. Thus, every ABM run identifies infected persons where they live and work in general, and their movements within groups (referred to as social networks) that influence disease spread. ABMs have been used to describe phenomena from social systems to immune systems, both of which are distributed collections of interacting entities (agents) that function without a leader. Simple agents interact locally according to simple rules of behavior, responding in appropriate ways to environmental cues and not necessarily striving to achieve an overall goal. An ABM consists of a set of agents that encapsulate the behaviors of the individuals that make up the system, and model execution consists of emulating these behaviors [2].

#### 3. Models of Influenza Transmission

The major focus of the MIDAS research partnership has been pandemic influenza. In response to the MIDAS mission (see http://grants.nih.gov/grants/guide/notice-files/NOT-GM-06–106.html), a number of large ABMs describing influenza transmission were developed. The main purpose of the models was to examine possible intervention strategies that would protect the general public from morbidity and mortality should an influenza pandemic strike. One of the initial objectives in all disease transmission studies is to determine the basic R0 value. The goal of intervention is to reduce R0 below the self-sustaining threshold of R0$\,=\,$1. The R0 of a future newly emergent influenza strain is unknown, but estimates for previous pandemics are available. For example, an estimate of 1.89 was obtained for the pandemic of 1968 in Hong Kong [3], and the pandemic of 1957 in Great Britain (GB) was estimated to be between 1.5–1.7 [4]. Also, the reproductive number of the first wave of the 1918 pandemic in the United States was estimated as 2–3 [5]. There is historical evidence that these three influenza pandemics were explosive. The results reported by the MIDAS also suggest that the pandemics can be controlled to some degree. By comparison, childhood diseases such as rubella, pertussis, and measles have R0 values in most populations in the range 7–15 [6], and consequently, are much less controllable.

To estimate the effect of various interventions on the spread of pandemic influenza, the effect of specific interventions on transmission rates needs to be quantified. Generally, estimates of the proportion of infections that occur in the various social contexts such as households, schools, workplaces, and communities have been reported. Then, estimates of relative effect of intervention measures on transmission in each context will need to be established. It would also be useful to know how implementation of a particular measure might disrupt contact patterns in other social contexts. For example, we would like to know the extent to which household and community contacts are increased when schools are closed.

There is scant information on the proportion of transmission that occurs in different social contexts. The best data available only allow the proportion of transmission in households to be quantified [7], [8]. Therefore, while models can give some insight into the likely benefit of single or combined interventions, that insight is somewhat limited by this lack of data. To some extent, the results depend on the assumptions made by the modelers about transmission in the different contexts. The degree of uncertainty does depend on the specific control measures. Modelers can arguably better project the possible effects of antiviral and vaccine use, case isolation, and household quarantine than the effects of school closure, mask use, banning of mass-gatherings, or nonspecific social distancing measures.

The most recent MIDAS study examined the effectiveness of a set of proposed targeted, layered containment strategies that combined a number of transmission interventions currently available to public health planners in the United States [9]. These include nonpharmaceutical social distancing measures and antiviral treatment and prophylaxis. All three MIDAS models examined the same set of interventions, although each of the three implemented the interventions using different approaches. The three sets of models also examined the sensitivity of the effectiveness of the intervention combinations to thresholds for triggering the interventions, levels of case ascertainment and compliance, and the transmissibility of the circulating pandemic strain. The intervention scenarios examined reflect those being considered now by the United States Homeland Security Council and Department of Health and Human Services (DHHS).

### B. Model Repository

An important goal of MIDAS was creating a repository for storing and managing the computerized models, model results, model parameters, and the specifications used to develop the models. The MREP was developed to house the code being developed by the MIDAS research groups into a professional, organized, and controlled environment. The guiding premise is that responding to an emergency requires a process that can be activated in a controlled, orchestrated manner; this premise also allows legacy code to be identified and past results reproduced. The MREP needs to fit into and support the emergency response process. To support ease of access, the MREP is implemented using relational database management technology and a Web-based interface.

In addition, there are other compelling reasons for developing the MREP, including promoting quality assurance and enhancing productivity during day-to-day activities. Losing track of the exact versions of the model that generated specific model results is easy. By imposing a structure that links together all model components, houses these connected components in a centralized database, and annotates those components, we can preserve and reuse essential linkages.

In summary, the MREP provides the following capabilities to the MIDAS: first, a process for responding to an emergency event. Characteristics of MIDAS models across all modeling groups can be quickly identified and linked to relevant documentation. Second, a quality assurance mechanism for registering models into the MREP. For a model to be part of the MREP, it must: 1) have a name; 2) be linked to a readily available description; 3) be linked to a contact; 4) identify the date and time of creation; and 5) specify the terms of distribution. The registration system is consistent with the process identified by Le Novère [10]. Third, a tracking mechanism that catalogs, locates, and identifies the different versions of the models involved in different experiments that comprise MIDAS studies. Fourth, the MREP has productivity features that allow modelers to efficiently locate previous model versions and reuse the code in new models. Fifth, the MREP maintains inventories of work developed by the research groups in a locatable form.

#### 1. MREP Scope

A repository is a collection of resources that can be accessed to retrieve information. Repositories often consist of several databases tied together by a common search engine. The MREP consists of a collection of infectious disease models and pertinent information about those models including: model specifications, inputs (static), parameters, results, source code, object code, scripts for compiling the object code, scripts for executing the model, user manuals, and other model documentation including published manuscripts.

Each element in the MREP is part of a relational database. This permits them to be linked to each other so that the inputs to a specific model and the results generated by running that model using those inputs are connected. Therefore, one major feature of the MREP is tracking and connecting all of the components of a model, which allows researchers to revisit previously generated results. A second important feature is that the linked components can be unified as part of the information retrieval process. This enables a query engine to present information to a user without having to browse irrelevant information.

In summary, the MREP is important to the MIDAS scientific research environment because it links specific model runs with the explicit model code and input data with the corresponding output data. It also provides a link to the specifications that guided model development. If researchers wish to identify how specific interventions were implemented and rerun a particular model or analyze it in any way after the run is completed, the MREP provides all the information necessary.

### C. Other Computerized Model Repositories

The MIDAS MREP appears to be unique among computerized epidemiological model repositories. However, a number of quantitative biology-based model repositories exist and provide a useful point of comparison.

We performed a literature search and identified other model repositories similar in some degree to the MREP. Specifically, we sought applications that maintained a database where models (model code, data inputs, and outputs) were shared by users in a controlled environment (i.e., an environment that connects model components). We identified a number of existing relevant model repositories and we contrast five of those that catalog and share information about a specific set of models and model runs. A number of online archives and/or data repositories from a number of nonmodeling applications were also identified.

The repositories described later offer information about a specific class of models to their user communities, and, in this context, they are similar in their capabilities to the MREP. None of them, however, catalogs infectious disease models and none of them attempts to maintain a version-controlled environment that offers code to the users from a stable, documented environment provided by a version-managed system. Nevertheless, all of the examples offer their users annotated models, and all are concerned about providing reliable programs with usable documentation.

#### 1. Biomodels Database

The BioModels.net project describes itself as an international effort to: 1) define standards for model curation; 2) define vocabularies for annotating models with connections to biological data resources; and 3) provide a free, centralized, publicly accessible database of annotated computational models in Systems Biology Markup Language (SBML) and other structured formats [11] (for detail, see http://www.ebi.ac.uk/biomodels/).

The database component of BioModels.net is especially designed for working with annotated computational models: each model is carefully reviewed and augmented by human annotators on the BioModels.net team to add metadata linking the model elements to other biological databases and resources. The BioModels database at the European Bioinformatics Institute (EBI) system is a true database, featuring browsing, cross-referencing, searching, and facilities for visualization, exporting models in different formats, and remote application programming interface (API) access.

The BioModels Database is a data resource that allows biologists to store, search, and retrieve published mathematical models of biological content. Models present in the BioModels Database are peer reviewed, annotated, and linked to relevant data resources, such as publications, databases of compounds and pathways, controlled vocabularies, and similar items. All models use SBML as their standard form of representation.

The premise of this tool is that researchers must be able to exchange and share their results. The development and broad acceptance of common model representation formats such as SBML is a crucial step in that direction, allowing researchers to exchange and build upon each other's work with greater ease and accuracy.

To make assembling useful collections of quantitative models of biological phenomena easier, establishing standards for the vocabularies used in model annotations as well as criteria for minimum quality levels of those models is crucial. The BioModels.net project aims to bring together a community of interested researchers to address these issues.

#### 2. CellML—A Biological Model-Based Repository

This application is similar to the BioModels Database application and uses a markup language called CellML developed specifically for describing biological processes contained in CellML [12]. The repository is a Web site that stores and exchanges computer-based mathematical models. This site allows scientists to share models described by the CellML markup language. It also enables them to reuse components from one model in another, thus accelerating model building (for detail, see http://www.cellml.org/examples/repository/index.html).

The CellML language is an open standard based on the Extensible Markup Language (XML) and is designed to describe biological models. The CellML also includes information about model structure (how the parts of a model are related to one another), model mathematics (equations that describe the underlying biological processes), and metadata (additional information about the model that allows scientists to search for specific models or model components in a database or other repository).

#### 3. AnyBody—A Repository for Models That Describe Musculoskeletal Processes

The premise behind this MREP is taken from the AnyBody Web site [13]. The site describes how the cost of musculo-skeletal injuries is rapidly increasing, while fundamental understanding of the mechanical functions of the body is increasing at a dramatic rate. However, it notes that there are still many unknown questions and problems researchers are addressing. For example, the AnyBody Web site indicates that the number of ergonomic-based injuries caused by excessive use of the computer mouse is exploding, yet the actual cause of many of these injuries remains a mystery. Policymakers and others, therefore, find it difficult to issue guidelines to reduce the problems. Also, research into human locomotion has historically been relegated to experimental studies (for detail, see http://anybody.auc.dk/).

The stated purpose of the AnyBody project is to develop mechanical models of different elements of the human body, and then, perform detailed studies of the behavior of these models. Typical model examples include the analysis and optimization of tools and workplace layout, and designing sports equipment and hand tools for maximum efficiency.

A unique software system called the AnyBody Modeling System was developed to conduct necessary research into causes and treatments of musculo-skeletal injuries.

There are four major aims of the AnyBody project. The first is to develop methods for analyzing movement strategies and tendon, muscle, and joint forces in humans performing specific manual tasks. The second is to investigate what numerical simulation can teach us about the function of the human body. The third is to use the analysis for ergonomic optimization of tools, workplaces, and man/machine interfaces. The fourth is to provide an MREP to enable interested researchers to share the models. Overall, AnyBody identifies useful information and models that can be shared by researchers interested in studying musculo-skeletal injuries.

#### 4. Probabilistic-Logical MREP

This repository contains software for manipulating and learning probabilistic-logical models [14]. The aim is to construct an MREP that will allow dissemination of software for probabilistic-logical models and facilitate comparisons among competing approaches (for detail, see http://www.informatik.uni-freiburg.de/$\sim$ kersting/plmr/).

The site notes that probability models are important methods for representing uncertainty, and mentions that various probabilistic frameworks include Bayesian networks, hidden Markov models, and stochastic context-free languages, along with other popular tools for describing appropriate scenarios exhibiting uncertainty. It notes that these types of models have been applied to problems in diagnosis, forecasting, automated vision, sensor fusion, manufacturing control, speech recognition, and computational biology. However, traditional approaches have a major drawback—they have a rigid structure, and therefore, lack of versatility in representing complex models.

To overcome these limitations, the site states that various researchers have recently proposed logical extensions of classical probabilistic models incorporating the notions of objects and object interconnections.

This repository consists of a database of software, documentation, and links to developers. The models are not specifically designed to be general purpose; rather, they are instructional in nature and represent a basis for discussion. The potential scope for the models in this repository is huge, with many different methods for defining and describing probabilistic-logical models.

#### 5. Cancer Intervention and Surveillance Modeling Network (CISNET)

The cancer intervention and surveillance modeling network (CISNET) is a consortium of investigators sponsored by the National Cancer Institute “whose focus is using modeling to improve our understanding of the impact of cancer control interventions (e.g., prevention, screening treatment, etc.) on population trends in incidence and mortality” [15]. They use models to project future trends and help determine optimal cancer control strategies (for detail, see http://cisnet.cancer.gov/about/).

The CISNET also describes using a comparative modeling approach in which each modeler focuses on an individual area. However, whenever possible, they develop a common “base” question that allows comparison across all models using a set of common population inputs. Then, they develop a common set of intermediate and final outputs.

To aid in this process of model description and comparison, the CISNET has developed the Model Profiler, an Internet-based application. Each CISNET team has a private model profile Web site on which it maintains model profile information and controls what parts of the profile are shared with other teams. By using a core documentation format that is the same for each group, the published profile information can be compared among models. The system allows modelers to describe their models, and allows interested readers to read about, compare, and contrast simulation models.

The sites described earlier we believed were fairly representative of the repositories available to researchers. Our literature search indicated that other sites are available. SigPath is described by Campagne [16] as an information management system that stores both quantitative information on cellular components and their interactions, and the basic reactions governing those interactions; EcoCyc, which is described as a comprehensive database resource for *Escherichia coli* by Keseler [17]; JWS Online, described by Olivier and Snoep as a repository of kinetic models describing biological systems that can be interactively run and interrogated over the Internet [18]; and the Database of Quantitative Cellular Signaling, which Sivakumaran describe as a repository of models of signaling pathways [19].

### D. Summary

A number of model repositories are identified in the literature. Many of these models support computational biology applications, and certainly, by some measures are more sophisticated than the MREP we describe here. One manifestation of sophistication is using a markup language based on XML concepts that imposes a standard method for describing and representing models common to a repository. We assert that ABMs are conceptually less mature than computational biology models, and broader in their scope (i.e., agents can represent genes, proteins, and *cis*-regulatory elements at one end of the spectrum, and represent people, states, and countries at the other end). We chose not to grapple with the notion of trying to develop a common taxonomy to describe agent-based epidemiology models. Rather, we focused on building a repository that captures ABMs in whatever form they were developed and creates a common set of documentation and annotations, so that the model can be understood (at some level) and reused should the need arise.

## II. Methods

### A. Scope

The MREP is designed to house, manage, and allow users to run infectious disease models from an access-controlled disease MREP. The MREP contains source code of disease models that have been developed by external developers and tested in a production environment. Different versions of models used to describe various aspects of the same disease are housed in the repository. During registration, models are linked to their developers, to a paper or PubMed reference that describes the model, to the name and contact information of model creators, to the date and time of creation, and to the terms of distribution. In addition, a code, available from the versioning software described later, is used to distinguish between different model versions. The annotations captured during the model registration process also identify a model's purpose, specifications, and relevant features.

The MREP also houses, manages, and controls access to a disease model results warehouse, which consists of output generated by the models contained in the MREP database. The results, tables, and files will be linked to the version of the model and the input parameters that collectively generated them. They will also be stored in a relational database to permit them to be easily identified, categorized, and downloaded.

The MREP includes a version control system (also referred to in the literature as a configuration control system) as one of its core elements. The system manages the source code, documents, graphics, and related files. Version-control software provides a database that is used to keep track of the revisions made to a program by all the programmers and developers involved in it.

The MREP uses Subversion as its version control system [20]. Subversion is a free, open-source application that is licensed under the Creative Commons Attribution License (see http://svnbook.red-bean.com/en/1.0/). The MREP also includes a Graphical User Interface (GUI) application that allows the user community to use the MIDAS models more easily. The GUI interfaces with Subversion, the database management system, and the computer environment. The GUI allows the user access to the model source code, provides an interface to input data sets, permits results to be viewed directly or downloaded to a user workstation, and provides a mechanism to submit the models for rerunning.

The MREP is comprised of the following major components: 1) a source code repository and version control system; 2) a model documentation tree; 3) a data warehouse (input and output data sets); and 4) an application interface (API) consisting of a database, browser, and graphics user interface components that allow the model user to develop input data sets, run the codes, and browse output results.

A standard system development approach was used creating the initial version of the MREP using approximately two full-time equivalents (FTEs) over a 13-month period.

### B. Architecture

#### 1. Overview

There were two features that were considered extremely important by the developers to include in the MREP design. These features include: 1) a version control system that provides configuration control over model source code and 2) complete flexibility regarding output formats. The first of these features was a response to an absence of model standards (such as SBML) in the storage of source code. The most common model development languages represented in the MREP are anticipated to be C, C$++$, Java, and Matlab. To maintain control in a language-free environment, using a version control element was deemed essential.

**Fig. 1.**
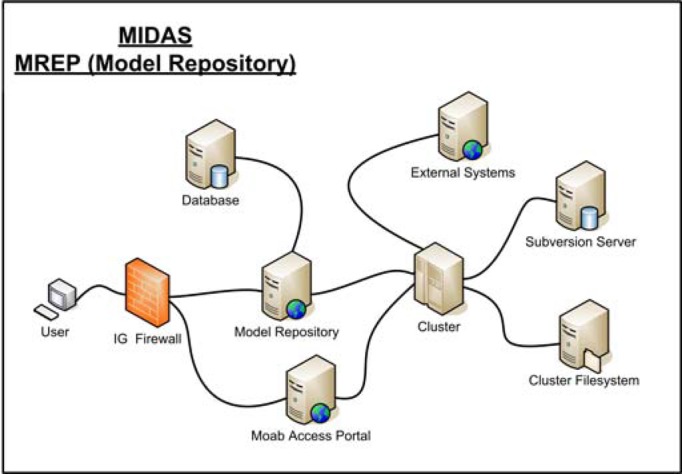
MREP Overview.

The second feature was the ability to support a common output format. All of the models generate results in different formats, including Concurrent Versions System (CVS), text, and Portable Document Format (PDF), among others. The MREP offers these results to users in the received format. Users can view results directly or by downloading a file and using a viewer that they supply. Fig. 1 represents a high-level logical data model for the MREP. Five primary components comprise the architecture, including the MIDAS Compute Server; a File System; a relational database management system (RDBMS); a Version Control System; and an External Systems Gateway. A description of each is provided later.

#### 2. MIDAS Compute Server

The MIDAS cluster is the central computational resource for the MIDAS research groups and is referenced in Fig. 1 as the cluster. The primary interaction that an analyst using the system will have with the cluster is through interacting with the job queuing system. Additionally, code maintainers/developers will have full access to all of the Linux system's features as needed to make changes to the code.

The MIDAS cluster is managed by Cluster Resource's MOAB (see http://www.clusterresources.com/pages/products/moab-cluster-suite.php) [21], an advanced cluster scheduler capable of optimizing scheduling and node allocations. MOAB allows site administrators to control job scheduling, priority, and where jobs are run.

#### 3. File System

The file system is part of the MIDAS cluster resource and is an integral part of the cluster system. A second system element is primarily used to archive study results in the MREP warehouse. At present, the archival file system is configured with 2 terabytes (TB) of disk storage that will eventually be expanded to 14 TB. Users interact with the file system to associate simulation run I/O data locations with metadata that will be used to identify and tag achievable simulation results. The configuration control system also interacts with this system.

#### 4. Relational Database System

The database management system platform will be housed by the MIDAS Web portal, which serves as the interface for electronic information exchange for the MIDAS network. The MIDAS portal is accessible to the public, but only registered users can access and provide information to the private section of the portal.

The MIDAS portal runs on RTI's Oracle Application Server 10 g (v. 9.0.4.1.0) server farm and uses Oracle technology [22] to manage the information within the repository (see www.oracle.com/appserver/index.html).

The database system tags metadata that reference specific simulation run results with the input and output data sets and source code associated with those runs. The database system will allow users to perform keyword searches to identify repository elements assigned to those metadata.

##### a. Data model—hierarchal design

The database design will maintain tables of metadata that describe the following entities:

Projects—a collection of studies with a common set of objectives; Studies—a collection of runs that were produced by one or more models; Models—the core code that is designed to describe computer environments that generate the runs; Model versions—a specific instance of a model to handle the production of runs having a specific set of attributes; and Runs—a set of information referred to as results that describe a single realization of a simulated epidemic.

Each realization is associated with a unique set of parameter values or alternatively is associated with a repeated set of parameter values. In the last situation, the results are referred to as a run replicate. Each model run produced by a specific model version is housed in the results data warehouse and consists of information that is part of the experiment.

**Fig. 2.**
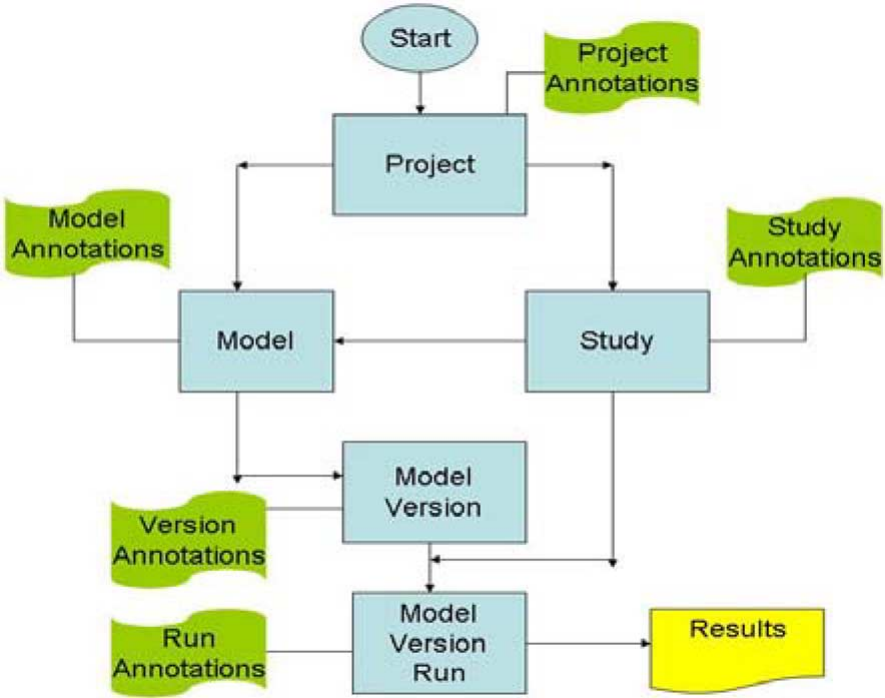
MREP Data Model.

Fig. 2 defines the interconnections between the entities. A study is either linked to a published manuscript that defines project aims and study results or to a document that describes an MIDAS study that is part of a larger MIDAS project effort to examine specific hypotheses about disease spread and containment. For example, the DHHS project examined the problem of whether influenza could be stopped at its source (i.e., South East [SE] Asia), and if so, what methods of containment are important. Two studies from this project are part of the MREP: the Emory SE Asia Study and the Imperial SE Asia Study.

In a second example, the combined project focus is to address the problem of “what can be done to mitigate pandemic influenza if it gets established in the U.S.” The specific objectives of the combined study are to assess the feasibility and effectiveness of different types of interventions strategies. The types of interventions specifically exclude prepandemic vaccines and limit the available quantity of a partially efficacious vaccine, but utilize as much antiviral treatment as required. Two paradigms are the focus of this study: the entire United States and a large city (Chicago). Six studies (and corresponding models) examine this problem. Two of these models simulate transmission in the continental United States, three others represent transmission in the city of Chicago, and the sixth presents the results of a historical review of the 1918 Spanish influenza epidemic. The last study helped justify the design of the intervention strategies.

Each model is composed of one or more model versions. Usually, a modeler attempts to describe the complete set of simulations through parameter manipulation. However, in many instances, it is likely that new and novel interventions will not be anticipated by the code developer. In these instances, it is common for the developer to create a different version of the model to handle a subset of the simulated interventions. One of the explicit objectives of the MREP is to track the different model versions and to link them to the results they produce.

The run is the lowest unit of analysis of the MREP. Each run constitutes a single replicate of a single set of parameters or a summary run over all replicates having a common set of parameters.

##### b. Query engine

The overall plan is to populate the MREP with models that are initially in accordance with MIDAS goals, which are currently limited to infectious disease models. Eventually, we anticipate including models from a broader perspective; namely, models that are relevant to any pathogen and appropriate transmission environment. This will potentially necessitate using a query tool to readily identify models of interest. For example, the MIDAS ABMs use synthetic population data that describe a specific geographical region. The model results identify individuals that influence disease spread within that region. The query tool enables users to identify a set of model results that pertain to a region of interest. Then, by using the geospatial identifiers that are tagged to individual model results, users can drill down into subregions and neighborhoods that are affected by epidemics.

Please note that it is possible to use the search keys to identify the studies that focus on a particular type of intervention strategy, and then, by examining Model Version details, determine the implementation details to decide a target computer to rerun the model. This could be done to replicate earlier results or to begin the process of modifying parameters to assess new interventions.

#### 5. Version Control

The version control system maintains the various model version source codes and executables. Each model version in the MREP is maintained in Subversion, the free, open-source version control system that manages files and directories over time. A tree of files is placed into a central database. This database is similar to an ordinary file server, except that it remembers every change made to files and directories. This allows the user to recover older versions of data, results, and/or code and to examine their change history.

Subversion is a distributed application; therefore, it can access its database across networks. This allows people to use Subversion on different computers and fosters collaboration by allowing various people to modify and manage the same set of data from their respective locations. Furthermore, progress can occur more quickly because there is no single conduit through which all modifications must occur. Because the work is versioned, we prevent the possibility of losing that conduit if an incorrect change is made to the data, in that the change can be easily undone.

Subversion is a full version control system. The MREP only uses a small subset of Subversion's functionality. The MREP user interface allows registered users to check out model executables that the user may then run on the RTI cluster. Another MREP interface allows the user to browse the source code tree for any model of interest. The MREP Subversion server is hosted on a virtual Linux host using VMWare Server software.

#### 6. External Applications

An important component of Fig. 1, referred to as External Systems, is a general set of tools that are available outside of the MREP. These tools are used to visualize, process, and analyze results from the MREP. The data from the MREP are served up via a HyperText Markup Language (HTML) portal. These data can be downloaded to the user's workstation or can be visualized directly from the MREP. For example, many of the data files in the MREP are stored as PDF, text, or.xls files, and can be viewed directly from the repository using Adobe Acrobat Reader, a text editor, or Microsoft Excel, respectively. Other files can be downloaded and imported into external systems available to specific users.

### C. Model Registration

The results/outputs of production runs will also be housed in the MREP. The model results will only include outputs from registered models. For a model to be registered, it must be placed under version control.

#### 1. Source and Object Code

When output from the model is used in a paper submitted for publication or otherwise presented publicly, or when the model's code is declared stable (by the developer), the model is a candidate for inclusion in the repository. If it has not been developed under version control, it will be moved to Subversion, tested, and moved to the MREP data warehouse.

The model code will be annotated with the following set of metadata: name of model, link to model description, contact information, date of model creation, distribution terms, model specifications, model implementation of those specifications, disease, region of analysis, types of intervention strategies, computer resource requirements, user's manual (link), validation measures (link to supporting manuscript), and compile and/or run scripts. The information about model specification and how those specifications were implemented is particularly important for explaining model differences. For example, if an implementation strategy calls for a reduction of 50% in model contacts, it is plausible to implement the strategy by halving the number of people contacted or alternatively maintaining the same number of persons in the contact network while halving the number of contacts with each person. The model results for each of the implementations could vary significantly.

#### 2. Model Results

Model results are also captured in a model results warehouse and linked to the model that generated them. Each result unit is tagged to a second set of metadata that includes model name, version number, developer, intervention, parameter file, fixed input file, and script used to generate results.

#### 3. Model Inputs

Model inputs are also placed in the repository and linked to the model that uses the inputs and the corresponding results that are generated. Each set of inputs is tagged to a set of metadata defined at the model version level: model name, version number, developer, intervention, and parameter location (including name, type, and range of values).

### D. Information Retrieval

Either a query tool is used to locate repository results or a complete list of models is displayed, and the user selects from the list. These results are then available for download and display. The process proceeds according to the following five steps.

In step 1, query keys are specified: the study and/or model and/or results are selected that meet the user-specified search criteria. For example, disease, geographic description, objective of study, model name and version number, and model developer.

In step 2, the query tool identifies the model in the repository with the specified attributes and displays the information. At this point, the user can either access the annotations or drill down to lower-level (run-level) linked results (results are displayed and/or downloaded if desired), or identify input data files and scripts that run the model.

In step 3, the model identified by the earlier steps can be checked out of Subversion. Please note that all version updates are performed by the MREP administrator and that model development occurs under version control. When a model is modified, debugged, and tested, it can be entered back into Subversion, but only as new model version. The modified model is then entered into the repository.

In step 4, the model is loaded into the MREP. This step annotates the model with both descriptive text and model metadata as part of the check-in process. An HTML file is created that connects the model, results, and input data; it also creates directories for source code, object code, inputs, results, and scripts.

In step 5, model results are loaded into the model result warehouse and metadata specified at the run level, including model name, version number, developer, result category, replicate, link to parameter list, and location of results.

## III. Results

Currently, four projects, 12 studies, five models, six model versions, and 538 runs are loaded into the MREP.

The vaccine project is a single study project that uses a single influenza-based model of a medium-sized city in the United States. It was developed by the Emory Group headed by Ira Longini. The study consists of six runs generated from a single epidemiological model of disease spread. The main hypothesis behind the vaccine distribution study is to assess whether targeted antiviral prophylaxis (TAP), taken prophylactically, is effective in containing influenza. The authors conclude that TAP is nearly as effective as vaccinating 80% of the population, and further, that vaccinating 80% of children less than 19 years of age is almost as effective as vaccinating 80% of the entire study population.

The Influenza Containment—SE Asia was developed by the MIDAS network and was completed in September 2005. It consists of two studies and two models. Both studies simulated disease transmission in regions that included some part of Thailand. The Imperial SE Asia model was developed by Ferguson [23] and the Emory SE Asia model was developed by Longini [24]. The overall hypothesis of the project was to examine if it is possible to contain Avian Influenza in the place of origin before it becomes a pandemic. The Emory SE Asia study represents a region of rural Thailand and consists of one model and 18 runs. Sixteen of the runs were produced by a single version of the model. However, the developers used a special version (the second) of the model that simulates the impact of geographically targeted antiviral prophylaxis (GTAP) to produce two other runs. All runs are loaded in the MREP. The Imperial model represented all of Thailand plus a perimeter region around its border that extended into its four border countries. Only the baseline (no intervention) run, produced by the Imperial SE Asia model, is loaded into the MREP at this time.

The Influenza Containment—United States and Great Britain project examined whether pandemic flu could be mitigated in the United States, assuming containment in SE Asia failed. This project was developed in collaboration with the DHHS and consisted of three studies: the Imperial Assessment study, the Epicast assessment, and the EpiSims assessment. The Imperial Assessment study consisted of two models: one describing disease transmission in the United States and the second describing transmission in GB. The GB model served as a counterpoint for the United States model, suggesting some interesting influences of geography and national patterns of behavior on disease spread.

The Epicast DHHS experiment described disease in the United States and examined influences on the spread of disease on a population derived from the U.S. 2000 Census data. The principal investigator of the Imperial assessment study is Ferguson [4]. The MIDAS principal investigator of the Epicast assessment study is Ira Longini [25].

The EpiSims assessment study simulated disease behavior in a mid-size city in the United States and used the same type of containment strategies that were used by the other two U.S. experiments. The EpiSims DHHS experiment consisted of a single model and generated 516 runs, involving a complete factorial design of nine binary variables and a partial design that examined the influence of three more variables. A complete set of 516 runs is loaded in the MREP for this study. The baseline GB run is also loaded into the MREP. Four national U.S. Epicast runs are currently loaded in the MREP.

The Combined project is also a U.S.-based study. It represents a refinement of the Influenza Containment—U.S. and GB project. Specifically, it examines a more complete set of social distancing interventions with the goal of determining practical strategies for implementation at the state and local level. This is an ongoing study that consists of six sets of experiments: two U.S. experiments, three Chicago-based experiments, and a historic study that examined certain characteristics of the 1918 influenza pandemic. Each study is associated with a single model with a single version per model. The objective of the combined study is to simulate a set of scenarios at the city and the national level. The scenarios are designed to address specific concerns by various federal agencies. The entire study amounts to about 150 distinct scenarios, each with multiple (thousands of) runs. This is an ongoing study that will be loaded into the MREP. Currently, only the principle results are loaded into the MREP; the code that generated the results has not yet been loaded.

A set of economic runs has also been generated as part of this study. The economic models involve assessments of cost-effective interventions with respect to containing disease spread.

## IV. Discussion

The MREP represents a one-of-a-kind resource for housing and cataloging infectious disease models. The strength of the MREP's design is its data model hierarchy that accurately portrays the stages of a study and its derivatives, at least from the MIDAS perspective. This data model begins with a high-level study as represented by an overall set of study objectives and design specifications and culminates at a low level with a set of runs (results) that contribute to assessing those objectives. In between the studies and the runs linked to those studies are the experiments that represent the different and independent points of view characterized by different research groups and the models those groups used to generate their results. The final data model design element is based on the assumption that containment strategies are not always accommodated within a single model; a change in the model code is sometimes the favored approach for representing simulated behavior differences, that is, the containment strategy responses.

A second important element of the MREP is its use of a recognized code versioning application to house different model versions. Using Subversion fosters a highly controlled environment that promotes quality assurance/quality control (QA/QC) activities through a rigorous association among different model versions—their inputs and the results the input data set and model version generate.

A final feature of the MREP is the absence of standards associated with including models results. The MREP permits Statistical Analysis Software (SAS), text, Excel, images, and virtually any output format for which a reader exists. Results can be left in the source environment or downloaded onto a user's workstation.

In the future, we plan to add an epidemiology-based ontology and develop a separate query tool. This is in part a substitute for a markup language. This tool will identify all models within the MREP (using the ontology information) that reference specific model parameters and connect those parameters to the values assigned by the study developers. This will provide a convenient mechanism for identifying and comparing assumptions across models within the MREP.

## Supplementary Material

Color versions of one or more of the figures in this paper are available online at http://ieeexplore.ieee.org.
